# A single-synapse resolution survey of PSD95-positive synapses in twenty human brain regions

**DOI:** 10.1111/ejn.14846

**Published:** 2020-06-25

**Authors:** Olimpia E. Curran, Zhen Qiu, Colin Smith, Seth G.N. Grant

**Affiliations:** 1 Centre for Clinical Brain Sciences, Chancellor's Building, Edinburgh BioQuarter, University of Edinburgh, Edinburgh, UK; 2 Academic Neuropathology, Chancellor's Building, Edinburgh BioQuarter, University of Edinburgh, Edinburgh, UK; 3 Simons Initiative for the Developing Brain (SIDB), Centre for Discovery Brain Sciences, University of Edinburgh, Hugh Robson Building, Edinburgh, UK

**Keywords:** automated image analysis, human post-mortem brain tissue, postsynaptic density protein 95, regional diversity, synaptome atlas, synaptome mapping

## Abstract

Mapping the molecular composition of individual excitatory synapses across the mouse brain reveals high synapse diversity with each brain region showing a distinct composition of synapse types. As a first step towards systematic mapping of synapse diversity across the human brain, we have labelled and imaged synapses expressing the excitatory synapse protein PSD95 in twenty human brain regions, including 13 neocortical, two subcortical, one hippocampal, one cerebellar and three brainstem regions, in four phenotypically normal individuals. We quantified the number, size and intensity of individual synaptic puncta and compared their regional distributions. We found that each region showed a distinct signature of synaptic puncta parameters. Comparison of brain regions showed that cortical and hippocampal structures are similar, and distinct from those of cerebellum and brainstem. Comparison of synapse parameters from human and mouse brain revealed conservation of parameters, hierarchical organization of brain regions and network architecture. This work illustrates the feasibility of generating a systematic single-synapse resolution atlas of the human brain, a potentially significant resource in studies of brain health and disease.

## 1 Introduction

The vast majority of interactions between nerve cells occur at synapses. The spatial location and the molecular composition of the pre- and postsynaptic elements are important factors in their cellular function. More than half a century of anatomical, physiological and molecular research has established that synapses integrate the information that is inherent in patterns of neuronal activity that lead to output, that is behaviour. The postsynaptic elements of synapses are particularly interesting because they host multiprotein signalling complexes that link together neurotransmitter receptors, ion channels, signalling enzymes, adhesion and structural proteins, thus supporting signal integration and intracellular signalling (Collins et al., 2006; Fernandez et al., 2009, 2017; Husi, Ward, Choudhary, Blackstock, & Grant, 2000; Komiyama et al., 2002; Migaud et al., 1998).

A key component of these complexes is the scaffold protein PSD95, one of the most abundant synaptic proteins in the brain (Cho, Hunt, & Kennedy, 1992; Frank & Grant, 2017; Frank et al., 2016; Frank, Zhu, Komiyama, & Grant, 2017). The complexes formed by PSD95 have been extensively studied in vivo and shown to play a key role in short- and long-term synaptic plasticity and many innate and learned behaviours (Migaud et al., 1998; Komiyama et al., 2002; Fagiolini et al., 2003; Opazo, Watabe, Grant, & O'Dell, 2003; Carlisle, Fink, Grant, & O'Dell, 2008; Abbas et al., 2009; Feyder et al., 2010; Camp et al., 2011; Nithianantharajah et al., 2013; Ryan et al., 2013; Fitzgerald et al., 2015; van de Lagemaat et al., 2017). Importantly, PSD95 mutations in humans cause intellectual disability (Lelieveld et al., 2016; Moutton et al., 2018) and mutations in the proteins within PSD95 complexes are responsible for dozens of neurological and psychiatric diseases including schizophrenia and autism (Bayes et al., 2011; Fernandez et al., 2009, 2017; Feyder et al., 2010; Fromer et al., 2014; Grant, Marshall, Page, Cumiskey, & Armstrong, 2005; Husi et al., 2000; Pocklington, Cumiskey, Armstrong, & Grant, 2006; Purcell et al., 2014).

Mammalian synapses contain thousands of evolutionarily conserved proteins (Bayes et al., 2011, 2012, 2017), and these are differentially distributed in brain regions (Emes et al., 2008; Hawrylycz et al., 2012; Roy, Sorokina, McLean, et al., 2018; Roy, Sorokina, Skene, et al., 2018). Visualization of proteins at the individual synapse level reveals a far greater synapse diversity than could be anticipated from historical studies of synapse morphology, neurotransmitters and physiology (Micheva, Busse, Weiler, O'Rourke, & Smith, 2010; O'Rourke, Weiler, Micheva, & Smith, 2012; Zhu et al., 2018). Driven by the ability to systematically characterize the molecular composition of individual synapses across the whole brain, the term “synaptome” has been introduced to describe the catalogue of synapses in the brain or part thereof (Micheva et al., 2010; O'Rourke et al., 2012; Zhu et al., 2018).

We recently systematically examined the distribution of PSD95 and SAP102 (another postsynaptic scaffold protein in excitatory synapses) at single-synapse resolution in hundreds of subregions within twelve main regions of the adult mouse brain (Zhu et al., 2018). PSD95 and SAP102 were fluorescently labelled and tissue sections imaged with confocal microscopy; then, sophisticated image analysis tools exploiting computer vision and machine-learning were used to detect individual synapses and measure their molecular and morphological properties, classifying the synapses and mapping their spatial locations. Each brain region had a characteristic “signature” of PSD95-positive or SAP102-positive synapses, and synapses expressing both proteins had yet another map. When these molecular types of synapses were further subcategorized using data on synapse size, shape and intensity of labelling, it was possible to build a larger catalogue and show that each synapse subtype had a unique synaptome map. A striking feature of all these maps is that they reveal a 3D spatial architecture to the synaptic organization of the brain, termed the “synaptome architecture” (Zhu et al., 2018). We showed that synaptome architecture has the potential to store behavioural representations and was correlated with the structural and functional connectome. Mutations in *Dlg3* and *Dlg2*, which cause intellectual disability and schizophrenia in humans, respectively, caused a change in the PSD95 synaptome architecture, suggesting that synaptic diseases manifest with altered spatial distribution of excitatory synapse types (Grant, 2019; Zhu et al., 2018).

Understanding synapse diversity and synaptome architecture in the human brain will inform on many key issues in both basic neuroscience and neurological disease research, from how and when gene mutations exert their effects on particular synapses and circuits to whether the synaptome architecture of the mouse and human show conservation. As a first step towards creating a human synaptome atlas, we recognized the need to overcome several technical challenges. First, the need to develop robust molecular labelling of excitatory synapses in post-mortem brain tissue using immunohistochemistry (IHC) suitable for analysis with the SYNMAP pipeline developed for the mouse brain (Zhu et al., 2018). To optimize and validate our IHC labelling of PSD95, we took advantage of two lines of genetically modified mice that we had previously characterized: mice lacking PSD95 (*Psd95*^–/–^; Migaud et al., 1998) and mice expressing PSD95 fused to green fluorescent protein (PSD95eGFP; Zhu et al., 2018). *Psd95*^–/–^ and PSD95eGFP mice serve as negative and positive controls, respectively, for antibody staining. Second, given that the human brain is 1,000-fold larger than that of the mouse, we needed to explore the feasibility of acquiring single-synapse resolution data across the major brain regions. Although PSD95 immunostaining has been reported in specific human brain regions, including frontal cortex, caudate nucleus, putamen and hippocampus (Fourie et al., 2014; Glantz, Gilmore, Hamer, Lieberman, & Jarskog, 2007; Morigaki & Goto, 2015), there is no single comprehensive study of PSD95 distribution across a number of human brain areas at single-synapse resolution using a high-throughput quantitative analysis.

Here, we report a detailed qualitative and quantitative survey of the distribution and diversity of PSD95-labelled synapses throughout 20 human brain areas from four phenotypically normal individuals. We describe the PSD95 synaptome architecture of these human brain regions and analyse regional similarity within the human brain and between human and mouse brain. We discuss the utility of this data set for studies of the normal and diseased brain and the logistical considerations for large-scale human synaptome mapping.

## 2 Materials and Methods

### 2.1 Consent

Post-mortem human brain tissue was obtained from the Medical Research Council UK (MRC) funded University of Edinburgh Brain Bank (EBB). All procedures involving post-mortem human brain tissue were approved by East of Scotland Research Ethics Service (16/ES/0084). Informed written consent was obtained in relation to each subject.

### 2.2 Human subjects

Human brains from four control subjects (three males, one female; mean age 51 ± 6.9 (standard deviation) years) were used to image PSD95 using laser scanning confocal microscopy (LSCM). Details of the human subjects are listed in Table 1. None of the control subjects had a history of dementia, neurological or psychiatric disorders at the time of death. Human brain tissue donated to the EBB was processed according to departmental protocols. Briefly, formalin-fixed paraffin-embedded (FFPE) 4-μm tissue sections were stained with hematoxylin and eosin (H&E) for routine neuroanatomical and neuropathological surveys. Selected sections were stained with a panel of antibodies that detect phosphorylated tau for neuronal and glial inclusions, beta-amyloid for vascular and parenchymal amyloid deposition, ubiquitin for ubiquitinated aggregates and/or alpha-synuclein for Lewy body pathology. Gross anatomical and microscopic examinations of the brain tissue revealed only low age-related neuropathological changes in these control brains (Table 2).

### 2.3 Post-mortem procedures

At post-mortem examination, brains were macroscopically dissected into 1-cm-thick coronal slices and sampled according to the EBB protocol (Samarasekera et al., 2013). Small tissue blocks were immediately fixed in 10% formaldehyde for 24–72 hr prior to further tissue processing and embedding in paraffin wax. The blocks were sampled from 13 neocortical areas (six frontal, four temporal, one parietal, two occipital), one allocortical area (hippocampus), two subcortical grey matter areas (thalamus and caudate nucleus) and four infratentorial regions (midbrain, pons, medulla and cerebellum) (Table 3). The 13 neocortical areas are defined as Brodmann areas (BAs) and include sensory visual regions (BA17 and BA19), sensory auditory region (BA41/42), motor region (BA4), premotor region (BA6/8), associative frontal regions (dorsolateral BA9 and BA46, ventrolateral BA44/45, and orbital BA11/12), associative temporal regions (BA20/21 and BA38), associative parietal region (BA39) and associative occipitotemporal region (BA37). The pathological changes in neurodegenerative diseases are present throughout the brain parenchyma; however, careful selection of representative areas allows efficient screening of tissue for any potential disease entities (King et al., 2013). The choice of the anatomical regions examined was additionally guided by the availability of all 20 blocks for the four control cases. Tissue blocks were macroscopically dissected by experienced pathologists and the selection of blocks closely followed topographical brain anatomy using the patterns of sulci and gyri. Regions of interest were further identified using an atlas of the human brain (Mai, Majtanik, & Paxinos, 2015). Nissl-stained sections from blocks immediately adjacent (where possible) to those used for IHC were examined to assess and confirm the distinctive cytoarchitectural features of various areas. The absence of significant neuropathological findings was further confirmed by routine examination of immunohistochemical and special stains available from the Department of Academic Neuropathology, Edinburgh University.

### 2.4 Fluorescence immunohistochemistry

FFPE human brain sections (4 μm thick) were dewaxed and dehydrated as per the EBB neuropathology laboratory protocol. Briefly, sections were dewaxed in two 3-min xylene washes followed by four 3-min graded alcohol washes, comprising two 74°OP (IMS99%) alcohol washes and two 70% alcohol washes. This was followed by a 15-min wash in saturated alcoholic picric acid to remove any formalin sedimentation and a final wash in running tap water for 15 min. Antigen retrieval techniques were used to enhance cell surface staining, antigen detection from antigen-masked FFPE sections and reduction of non-specific background staining. The slides were placed in 250 ml freshly made antigen retrieval solution (0.1 M sodium citrate buffer pH 6.0, Fisher Scientific) and pressure cooked (A. Menarini Diagnostics) at 125°C for 30 s. After cooling in tap water, sections were washed once with PBS and blocked with 5% bovine serum albumin (BSA, Sigma-Aldrich) in 1x Tris-buffered saline (TBS, Fisher BioReagents) containing 0.2% Triton X-100 (Sigma-Aldrich) for 1 hr at room temperature (RT). Sections were then incubated overnight at 4°C with primary antibodies: PSD95 (IgG2a, mAb, K28/43, 1:250; NeuroMab), Synapsin1 (IgG, mAb, 13197S, 1:200; Cell Signaling), Synaptophysin (IgG, mAb, SP11, RM-9111, 1:200; Thermo), Calbindin D-28K (IgG, pAb, CB38, 1:50; Swant) diluted in TBS containing 3% BSA and 0.2% Triton X-100. After three 5-min washes with TBS and 0.2% Triton X-100, secondary antibodies (Alexa Fluor 546 goat antimouse IgG2a (γ2a; Molecular Probes) for PSD95; Alexa Fluor 633 (Molecular Probes) for synaptophysin or synapsin 1) were added at 1:500 and sections incubated for 2 hr at RT. Each IHC staining round was accompanied by nuclear cell counterstaining with 4′,6-diamidino-2-phenylindole (1 μg/ml DAPI (Sigma) diluted 1:1,000 in PBS). Finally, the slides were washed three times with TBS and 0.2% Triton X-100. For elimination or reduction of lipofuscin-like autofluorescence, sections were subsequently treated with Autofluorescence Eliminator Reagent (AER, Millipore Chemicon International). Lipofuscin pigment accumulates in the cytoplasm of brain cells and can hamper fluorescence microscopy owing to its broad excitation and emission spectra. AER is a commercially available Sudan Black (SB)-based reagent that has been successfully used in studies using human tissue (Blazquez-Llorca, Garcia-Marin, & DeFelipe, 2010). SB has been reported to provide the best compromise between the reduction of lipofuscin-like fluorescence and maintenance of specific fluorescent labels (Schnell, Staines, & Wessendorf, 1999). Briefly, the sections were first immersed in 70% ethanol for 5 min, followed by the AER for 30 s. Slides were washed in 70% ethanol for a further 3 min before being mounted with mounting medium (Mowiol with 1,4-diazabicyclo[2.2.2]octane (DABCO), Sigma-Aldrich) and left to dry overnight.

### 2.5 Validation of PSD95 IHC with PSD95eGFP mouse

Qualitative and quantitative comparisons with a selection of images from PSD95eGFP mice were made to validate the human PSD95 staining. At first, the representative IHC images of coronal sections from several mouse brain areas were acquired using the same LSCM and with the same parameter set-up. Then, a direct quantitative comparison of PSD95eGFP mouse versus human results was made for three wild-type (WT) male mice (aged 18 months) and three human male control subjects from fifteen brain regions. The PSD95eGFP mouse data were acquired with a spinning disk microscope and analysed using the ensemble analysis as previously described (Zhu et al., 2018).

### 2.6 Image acquisition

To resolve individual synaptic puncta, antibody-labelled brain tissue sections were captured using a Zeiss laser scanning confocal microscope (LSCM510) using a Zeiss Plan-Apochromat 63× oil-immersion objective lens (NA 1.4) with a frame size of 1,024 × 1,024 pixels, a pixel dimension of 46 × 46 nm and a depth of 8 bit. All raw images were obtained with 3.1× zoom as stacks of six image planes with *z*-step of 130 nm pixels and a total length of axial plane 780 nm pixels. The total area used for synaptic density determination varied depending on region, but for cortical areas 18 images, three images per each of six cortical layers, per BA per subject were acquired. The images taken were of neuropil, and structures, such as blood vessels or cell bodies, were avoided when possible. A total of 1,510 confocal images were acquired, of which 21 images (1.4%) were excluded due to technical problems, including too low signal-to-noise ratio precluding adequate image analysis, a lack of alignment in *z*-stacks and images with overexposed synaptic puncta. Images and figure montages were prepared in Adobe Photoshop and Adobe Illustrator. Where necessary, images were adjusted for brightness for correct display, but no other corrections were made.

For the visualization of the whole-mounted brain sections, a Zeiss Axio Scan.Z1 Slide Scanner with a 20× lens (NA 0.8) was used. Pixel resolution was 0.325 × 0.325 μm, and image brightness was in a depth of 16 bits. ZEN lite 2012 software (Zeiss) was used with the scanner for image acquisition and adjustment. Coronal brain section images were background and contrast adjusted to provide consistent comparison of PSD95 expression. All control images were directly comparable with their negative controls using the same settings. The slide scanner-captured images were used only for qualitative comparisons. The slide scanner was also used to capture Nissl-stained material for direct assessment of tissue integrity and cytoarchitecture of sections used in this study.

### 2.7 Image analysis

Analysis of the human synapse images includes synaptic puncta detection, segmentation and parameter quantification. The detection and segmentation of individual puncta were performed by ensemble learning, a machine-learning approach developed in house (Zhu et al., 2018). Ensemble learning of image detection requires an annotated image set from the LSCM data set for algorithm training. Raw tile images were first selected from the whole data set using random bootstrapping. PSD95-labelled individual synaptic puncta in each of the images were then manually annotated using the Fiji plugin “Cell Counter” (Schindelin et al., 2012) by two independent experts. Half of the annotated data were used for training and the other half for testing of the trained detector. A K folder strategy was also used for validation of the training. The second step in the ensemble image analysis involves algorithm training of the synaptic puncta detector, which is based on a multi-resolution image feature detection (Qiu, Lei, & Weiping, 2012) and supervised machine-learning technique. A multi-resolution and multi-orientation version of 2nd-order nonlocal derivative (NLD) to calculate intensity differences, or image features, was developed for each of all individual puncta. For instance, for an image of PSD95 IHC, a group of 109 feature images was calculated per punctum. These intensity differences were assembled as feature vectors of each individual punctum. Details of the algorithm training are provided in our previous work (Zhu et al., 2018). Once training was finished, the trained ensemble detector was applied to automatically detect puncta in the LSCM images. The detected object was then segmented by thresholding the pixel intensity of the puncta. The threshold was calculated adaptively for each punctum as 18% of the maximal intensity of the punctum after subtracting the background intensity.

Different puncta parameters were measured after the detection and segmentation. In particular, the mean punctum intensity is a measurement of the relative amount of PSD95 protein within the postsynaptic density (PSD), since the absolute number of PSD95 molecules cannot be resolved using confocal imaging. Nevertheless, the mean punctum intensity provides a good insight into the differences in average packing of PSD95 between areas or individuals. Puncta size is a measurement of the relative area of PSD95-positive synapses. It is calculated as the number of pixels within the segmented puncta and converted to μm^2^. The final measurement provided by ensemble image analysis is the puncta density, that is the number of detected puncta per 100 μm^2^.

### 2.8 Statistical analysis

Three PSD95 punctum parameters, namely density (puncta number per 100 μm^2^), intensity (A.U.) and size (μm^2^), were used for the statistical study. The parameters were plotted with the Tukey-style as box and whisker plots (Krzywinski & Altman, 2014). Normality of LSCM data distribution was analysed using the D’Agostino-Pearson omnibus normality test. For heatmap generation, the data were reorganized using an agglomerative hierarchical clustering algorithm and standardized using the *z*-score normalization. A similarity matrix in Figure 8a was generated to quantify the PSD95 synaptome network architecture in the human brain. Each row/column in the matrix represents a brain region/subregion. The rows/columns are resorted to indicate the brain anatomy: anatomically close regions are placed in adjacent rows/column in the matrix. The elements of the matrix are the similarity and calculated using the Euclidean distances of the three synaptic parameters between the two regions. It ranges from zero to one, the latter indicating an identical synaptome of the two regions. The similarity matrix was plotted as a heatmap where the elements were colour coded.

## 3 Results

The study is divided into four phases: (a) the optimization of synaptic labelling with antibodies to PSD95, (b) the systematic acquisition of image data from 20 brain regions with a detailed description of the synaptic anatomy, (c) an analysis of the populations of synapses in the brain areas and (d) a comparison of the PSD95 synaptome architecture of human and mouse.

### 3.1 Optimization of PSD95 immunostaining

**Figure 1:**
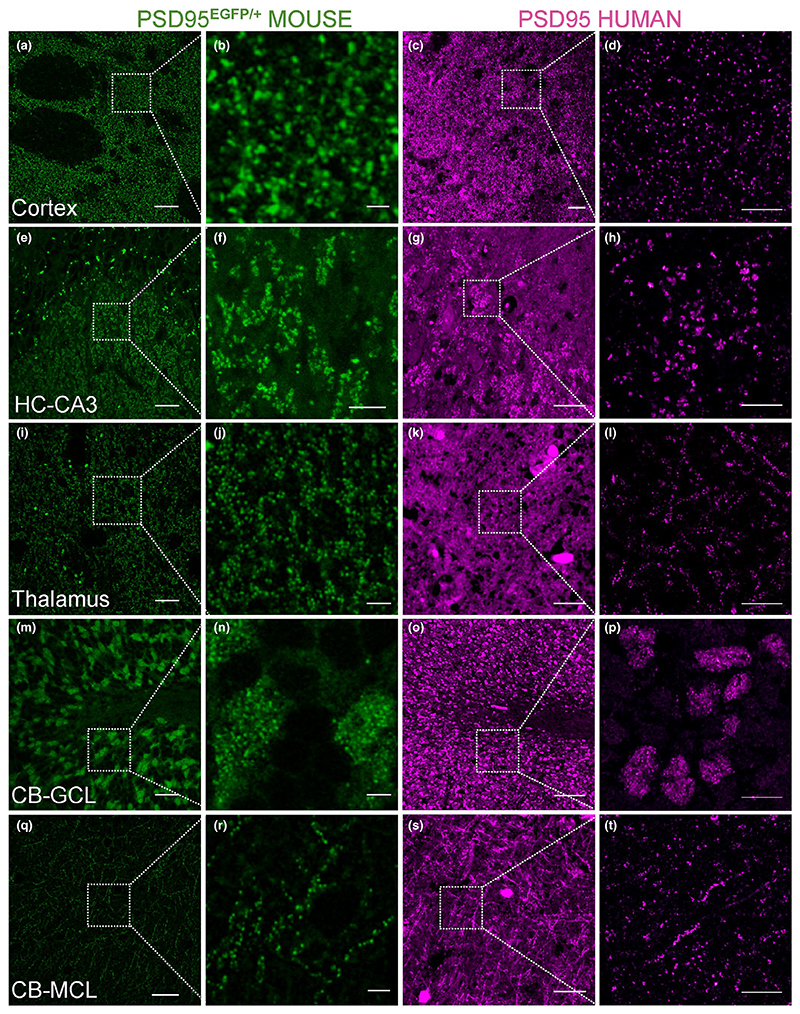
Comparison of PSD95 expression between human and mouse brain. Similarity of PSD95 labelling patterns between human and mouse. Panels in green: tissue from mutant knock-in mouse examined using direct fluorescence microscopy of PSD-EGFP fusion protein in the adult *Psd95*^EGFP/+^ heterozygote. Panels in magenta: human tissue stained with PSD95 antibody using indirect immunofluorescence. Similar patterns of staining were observed in human and mouse cortex (a–d), thalamus (i–l), cerebellar granular (m–p) and molecular (q–t) cell layers. Similar patterns of staining were also observed in the hippocampal CA3 region, although the staining was present in two different subregions: in mouse in stratum lucidum (e, f), but in human in stratum pyramidale (g, h). Scale bars: 20 μm in a, e, i, m, q; 2 μm in b, j, n, r; 5 μm in f; 30 μm in c, g, s; 100 μm in k, o; 10 μm in d, h, l, p, t

To identify optimal IHC conditions, we used four complementary approaches. First, we used the standard IHC controls including the presence and absence of primary and secondary antibodies on human tissue (Figure S1). Strong punctate staining was observed with PSD95 antibodies and abolished when either primary or secondary antibodies were absent. Second, to confirm that the PSD95-positive puncta observed were synaptic, we labelled the excitatory presynaptic terminals with synapsin 1 and synaptophysin (Figure S2; Koffie et al., 2009) and detected the characteristic juxtaposed labelling in 91.5% and 87.8% of synapses, respectively. Third, we performed PSD95 IHC on brain sections from Psd95 knockout mice and confirmed the absence of signal (Figure S3). Fourth, we compared the puncta distribution of multiple human brain regions with the corresponding regions in mice expressing PSD95eGFP, which revealed similar synaptic puncta distributions in these areas (Figure 1). We screened eight commercially available antibodies and found best results with anti-PSD95 monoclonal IgG2a (NeuroMab) at 16 ng/ml (a 250-fold dilution of the 4 μg/ml stock solution).

### 3.2 Human brain regions show different distributions of excitatory synapses

**Figure 2:**
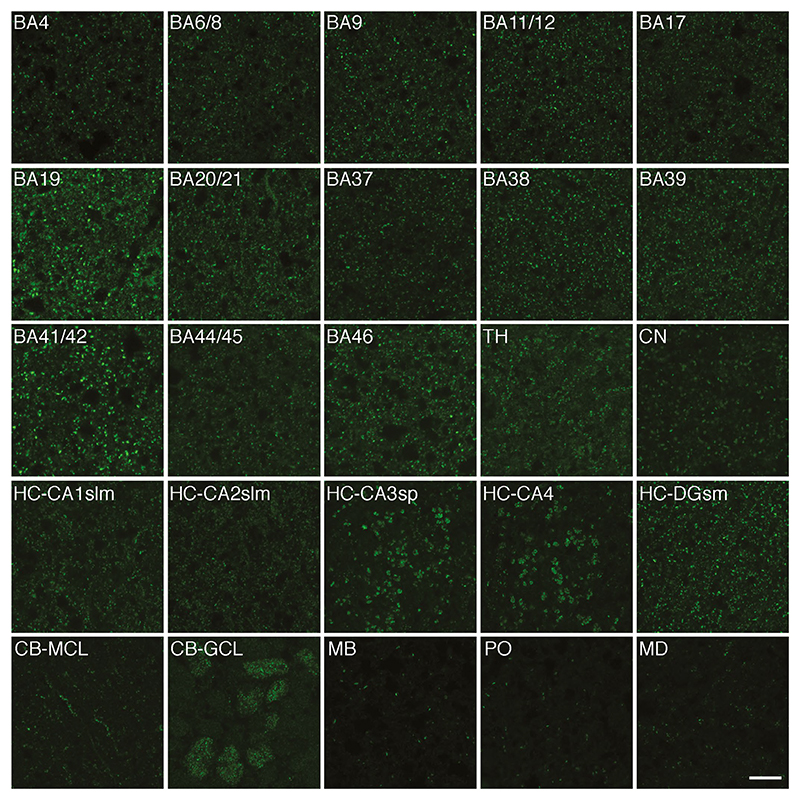
PSD95 expression in 20 human brain regions. Representative images from 20 human brain regions showing the different patterns of PSD95 distribution detected by immunofluorescence. BA, Brodmann area; CA, cornu ammonis; CB, cerebellum; CN, caudate nucleus; DG-sm, dentate gyrus stratum moleculare; GCL, granular cell layer; HC, hippocampus; MB, midbrain; MCL, molecular cell layer; MD, medulla; PO, pons; slm, stratum lacunosum-moleculare; sp, stratum pyramidale; TH, thalamus. Scale bar: 10 μm

In order to describe the distribution patterns of the PSD95-positive synapses in the human brain, we performed IHC on post-mortem material from 20 regions. Representative images from these selected brain areas are shown in Figure 2. PSD95 expression was detected as fluorescent puncta (<1 μm in size) in all regions. In general, the PSD95 puncta appeared intense and densely packed in the neocortex and the hippocampus. However, even within the neocortex, different areas displayed unique combinations of bright, dim, large and small puncta. In the subcortical structures, the thalamus and caudate nucleus, the puncta tended to show moderate densities and intensities. A high density of intense PSD95 puncta was found in some parts of the cerebellum, such as the granular cell layer, but not others, such as the cerebellar white matter. Both the hippocampus and cerebellum showed distinct subregional distribution patterns of PSD95 puncta, as described below. Synaptic puncta density was low in all major areas of the brainstem examined (midbrain, pons and medulla). Synaptic puncta were almost absent from the cortical and the cerebellar white matter (not shown). Thus, human brain regions show diversity in the densities, intensities and size of PSD95 puncta. In the following sections, we describe some of the diversity observed within the hippocampal formation, the deep grey nuclei (caudate and thalamus), cerebellum and brainstem.

**Figure 3:**
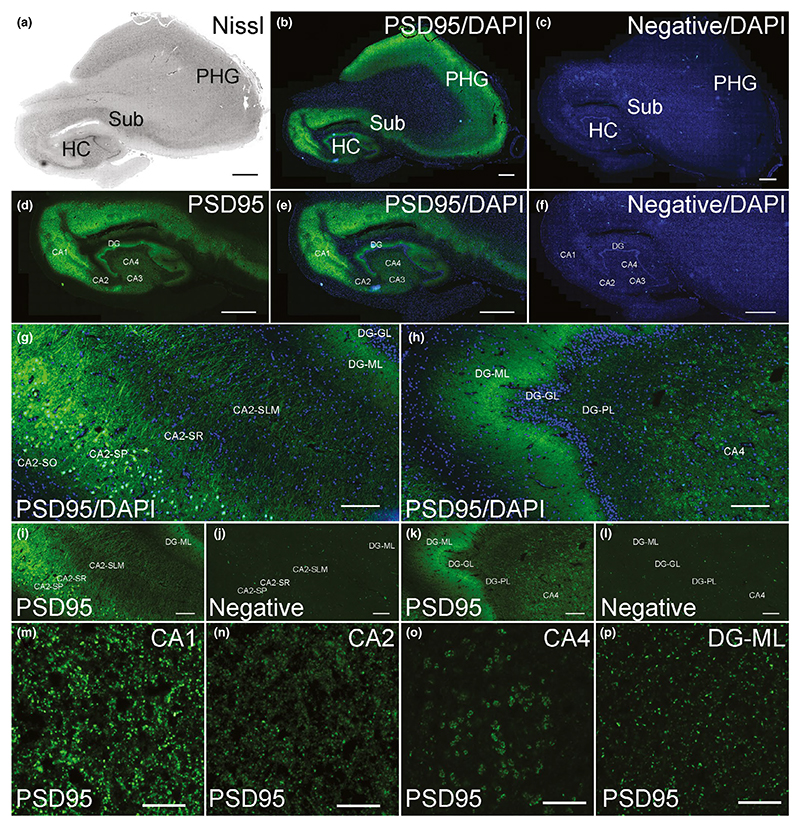
PSD95 staining in the human hippocampus. (a) Nissl-stained section assessing hippocampal neuroanatomy. (b) PSD95 labelling in the hippocampus, subiculum and adjacent parahippocampal gyrus. (c) Negative control stained without primary antibody showing absence of PSD95 signal. (d) PSD95 labelling, (e) PSD95 with DAPI and (f) negative control with DAPI in the hippocampus proper. (g) PSD95 labelling in the CA2 subregion. (h) PSD95 labelling in the DG subregion. (i) PSD95 labelling with DAPI, and negative control (j) in the CA2 subregion. (k) PSD95 labelling with DAPI, and negative control (l) in the DG subregion. (m–p) PSD95 staining in the indicated hippocampal subregions. DG, dentate gyrus; DG-GL, DG-granule cell layer; DG-ML, DG-molecular layer; DG-PL, DG-polymorphic layer; HC, hippocampus; PHG, parahippocampal gyrus; SLM, stratum lacunosum-moleculare; SO, stratum oriens; SR, stratum radiatum; Sub, subiculum. Scale bars: (a–f) 2 mm, (g–l) 200 μm, (m–p) 10 μm

In the human hippocampal formation, there was intense PSD95 staining within CA1-4 subregions as well as the dentate gyrus (DG; Figure 3a–c). The highest intensity of staining was seen in CA1 (Figure 3d–f). Bright PSD95 staining was mostly observed within the dendritic layers, including stratum radiatum and stratum lacunosum-moleculare (Figure 3g) as well as the molecular layer of the DG (Figure 3h,p). Staining was observed on the cell bodies of the pyramidal cells of the CA1-4 subregions (Figure 3i,j), in contrast to the granule cells of the DG that lacked staining (Figure 3k,l). CA1 contained small bright PSD95 puncta (Figure 3m), whereas CA2 puncta were dimmer (Figure 3n). The puncta observed in the neuropil of the pyramidal cell layer of the CA3 (not shown) and CA4 subregions (Figure 3o) appeared very similar in size, intensity and pattern of staining: they were large, moderately bright and formed a rosette-like pattern of staining.

**Figure 4:**
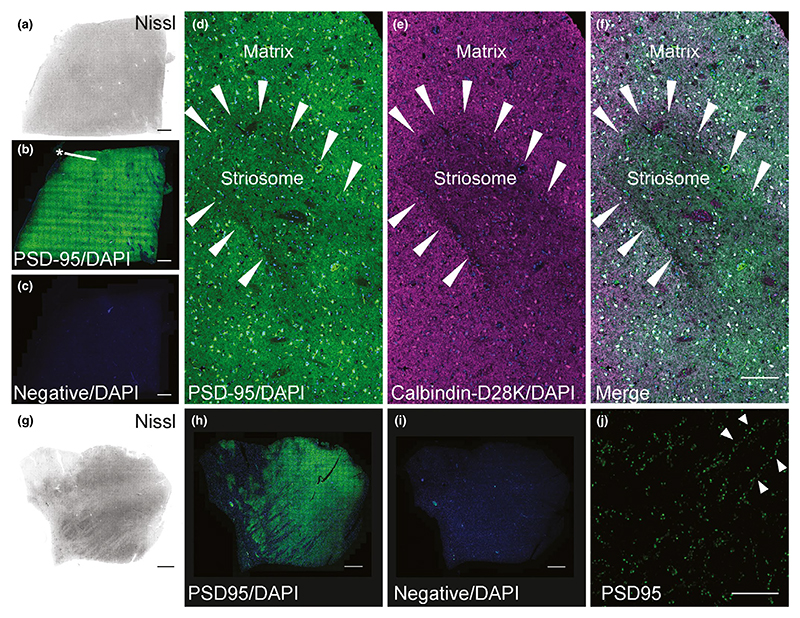
PSD95 staining in the human caudate nucleus and thalamus. (a–f) Caudate nucleus. (a) Nissl-stained section to assess neuroanatomy of the caudate nucleus. (b) PSD95 staining was not homogenous, as small darker patches of striosomes were identified scattered within the caudate matrix (asterisk). (c) Omission of PSD95 primary antibody showed a lack of PSD95 labelling. (d) PSD95 puncta within a striosome (arrowheads) and a matrix. Somatic staining of PSD95 in medium spiny neurons was present in addition to dendritic PSD95 puncta. (e) The same striosome visualized with Calbindin D28K antibody. (f) Merged image showing PSD95 and Calbindin D28K labelling. (g–j) Thalamus. (g) Nissl-stained section to assess thalamic neuroanatomy. (h) PSD95 labelling was variable, but prominent throughout the thalamus. (i) Omission of PSD95 primary antibody showed lack of PSD95 expression. (j) Punctate PSD95 staining along dendrites (arrowheads) within the thalamus. Scale bars: (a–c, g–i) 2 mm, (d–f) 100 μm, (j) 10 μm

The caudate nucleus showed strong PSD95 labelling (Figure 4a–c), with small patches (Figure 4b asterisk) that we confirmed to be striosomes by IHC with Calbindin-D28K (Ito, Goto, Sakamoto, & Hirano, 1992; Figure 4d–f), which are embedded within the matrix. In addition to dendritic staining, somatic staining was observed, presumably in medium spiny neurons. In the thalamus, moderate numbers of PSD95 puncta were detected (Figure 4g–i). PSD95 puncta were often found lined up along neuronal dendrites (Figure 4j), although a more diffuse distribution of puncta was also seen.

**Figure 5:**
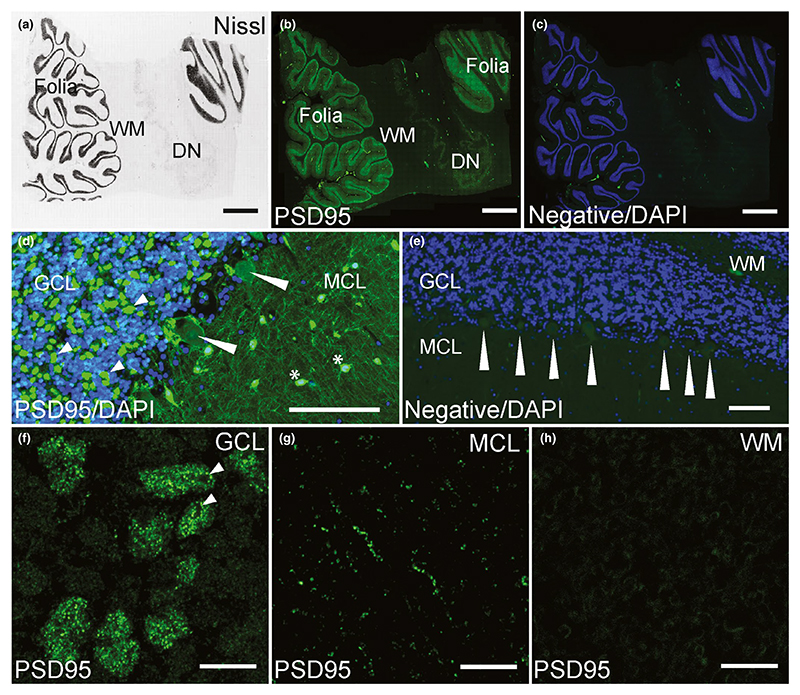
PSD95 staining in the human cerebellum. (a) Nissl-stained section assessing cerebellar neuroanatomy: cerebellar cortex (folia), WM and the DN. (b) PSD95 labelling in the cerebellar folia, WM and the DN. (c) DAPI with negative control stained without primary antibody showing absence of PSD95 staining. (d) Numerous and dense clusters of PSD95 puncta dispersed throughout the glomerular region of the GCL between the granule cells (short arrowheads). PSD95 puncta were detected in the terminal pinceau of cerebellar basket cells, but PC neurons did not express PSD95 (long arrowheads). In MCL, PSD95 was observed as fine linear pattern with strong labelling of stellate and basket cell nuclei (asterisks). (e) PSD95 staining abolished in a negative control section, showing lack of staining around and within PCs (arrowheads). (f) PSD95 staining in the GCL showing numerous intense puncta within the clusters. Arrowheads point to areas of rosette-like punctate formations. (g) PSD95 staining within MCL localized along dendrites. (h) No punctate PSD95 labelling was present in the cerebellar white matter. DN, dentate nucleus; GCL, granular cell layer; MCL, molecular cell layer; PC, Purkinje cell; WM, white matter. Scale bars: (a–c) 2 mm, (d, e) 100 μm, (f–h) 10 μm

In the cerebellum, PSD95 staining was restricted to the cerebellar folia, absent from the white matter and present at low levels in the dentate nucleus (Figure 5a–c). The adult cerebellar cortex is a three-layer structure composed of an internal granular cell layer (GCL), Purkinje cell layer (PCL) organized into a single row of Purkinje cells (PCs), and a molecular cell layer (MCL), and the pattern of PSD95 distribution varied between these layers (Figure 5d,e). Overall, PSD95 staining was higher in the GCL than in the MCL, and no PSD95 staining was seen in the PCs (Figure 5d,e). Numerous and dense clusters of PSD95 puncta were dispersed throughout the glomerular region of the GCL between the granule cells (Figure 5d small arrowheads). PSD95 puncta were only detected in the terminal pinceau (also known as the presynaptic plexus) of cerebellar basket cells, surrounding the axon hillock regions of PCs, but the postsynaptic PC neurons did not express the protein (Figure 5d long arrowheads). A much lower density of PSD95 was detected within the neuropil of the MCL, where staining within the larger nuclei of the stellate and basket cells was observed (Figure 5d asterisks). Higher magnification images highlighted the clusters of PSD95 puncta within the GCL between the granule cells, corresponding to mossy and climbing fibres, but the granular cells only showed non-specific staining (Figure 5f). A much lower density of PSD95 was detected within the neuropil of the MCL, where PSD95 puncta formed a fine linear pattern along dendrites (Figure 5g). PSD95 puncta were not present in the white matter (Figure 5h).

**Figure 6:**
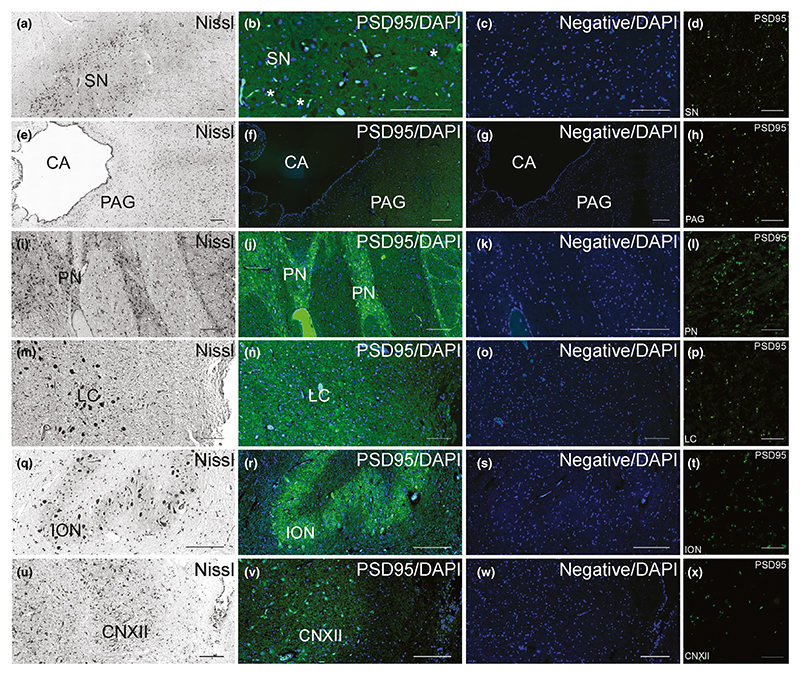
Pattern of PSD95 staining in the human brainstem. (a–h) Midbrain. Nissl-stained sections of the SN (a) and PAG (e) within the midbrain. PSD95 staining within the SN (b) and PAG (f) was of low density. PSD95 labelling was absent from large pigmented neurons of the SN (b, asterisk). No PSD95 expression was detected in the SN (c) or PAG (g) in the negative controls without primary antibody. Sparse density of PSD95 puncta in SN (d) and PAG (h). (i–p) Pons. Nissl-stained sections of the PN (i) and LC (m) in the pons. PSD95 staining within the PN (j) was low, but higher than in the LC (n). No PSD95 expression was detected in the PN (k) or LC (o) in the negative controls. Low density of PSD95 puncta in the PN (l), and sparse density within LC (p). (q–x) Medulla. Nissl-stained sections of the ION (q) and CNXII (u) within the medulla. PSD95 staining within the ION (r) was low, but higher than in the CNXII (v). Negative controls confirmed no PSD95 expression within the ION (s) and CNXII (w). Low density of PSD95 puncta in the ION (t) and sparse density within CNXII (x). CA, cerebral aqueduct; CNXII, nucleus of the cranial nerve XII (hypoglossal nucleus); ION, inferior olivary nucleus; LC, locus ceruleus; PAG, periaqueductal grey; PN, pontine nuclei; SN, substantia nigra. Scale bars: 100 μm, except (d, h, l, p, t, x) 10 μm

The midbrain (Figure 6a–h), pons (Figure 6i–p) and medulla (Figure 6q–x) were examined for PSD95 expression, and overall showed a sparser density of fluorescent puncta than the supratentorial structures. Within the midbrain, a low density of PSD95 puncta was present within the substantia nigra (Figure 6b,d), and a lower density was detected in the periaqueductal grey area (Figure 6f,h). The fluorescent signals in these areas were moderately bright (Figure 6d,h). Within the pons, moderate density of PSD95 puncta was seen in the pontine nuclei (Figure 6j,l) and low density in the locus ceruleus (Figure 6n,p). The fluorescent signals in these pontine areas were moderately bright (Figure 6l,p). Finally, within the medulla, a moderate density of PSD95 puncta was present within inferior olivary nucleus (Figure 6r,t), and a low density was detected in the hypoglossal nucleus (Figure 6v,x). The PSD95 puncta in these medullary areas were dim (Figure 6t,x).

### 3.3 Quantification of PSD95 puncta in human brain regions

**Figure 7:**
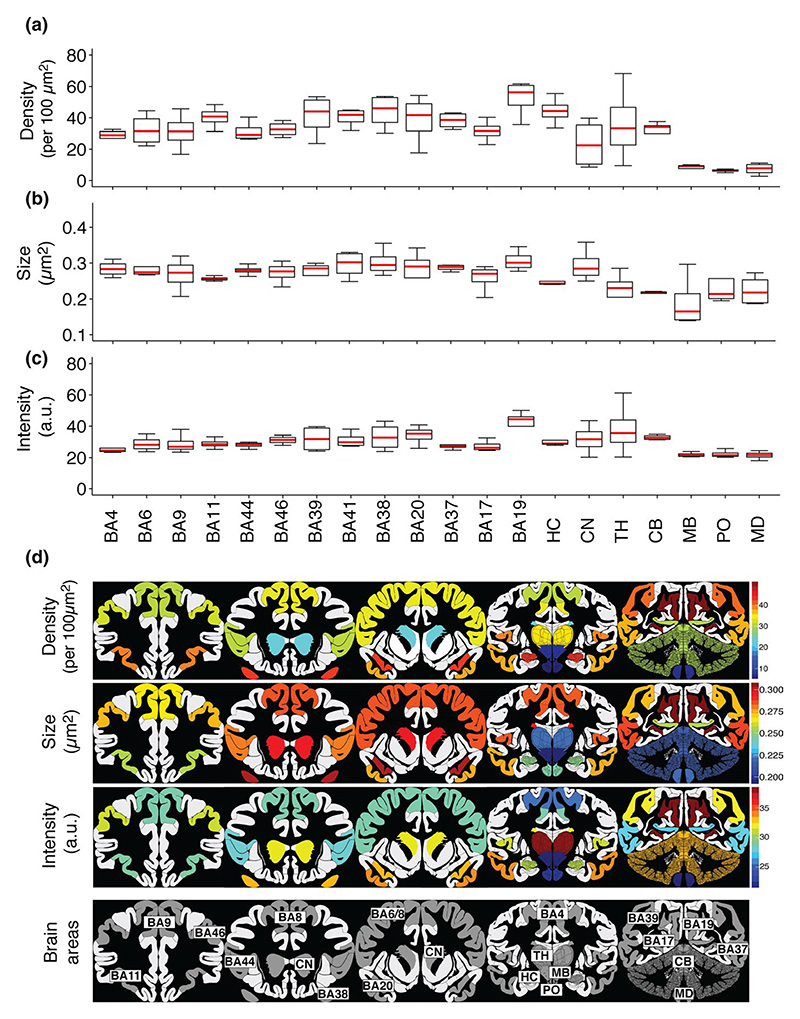
Quantification of PSD95 in 20 human brain areas. (a–c) Boxplots showing quantification of PSD95 puncta density (per 100 μm^2^) (a), size (μm^2^) (b) and intensity (a.u.) (c) in 20 human brain areas from four control cases. (d) Colour-coded PSD95 synaptome map based on quantifications of parameters. BA, Brodmann area; CB, cerebellum; CN, caudate nucleus; HC, hippocampus; MB, midbrain; MD, medulla; PO, pons; TH, thalamus

To quantify synapses and describe their diversity in the brain regions, we used the SYNMAP image analysis pipeline created to map the mouse synaptome (Zhu et al., 2018). Each PSD95 punctum was quantified in terms of its intensity (A.U.) and size (μm^2^). In addition, the density of PSD95 puncta per 100 μm^2^ was calculated as a mean of the number of puncta per region. Thevalues for four individuals are shown in Figure 7a–c, and the data from individual subjects are shown in Figure S4. Figure 7d shows a summary of the mean values of each of the puncta parameters on maps of the human brain and highlights the diversity of these regions.

Ranking the 20 brain regions shows that the highest synapse density is in the neocortex and hippocampal formation and the lowest density in pons, midbrain and brainstem. There was an 8.3-fold difference between the highest and lowest mean density in the examined areas. Cortical area BA19, a region of the occipital cortex involved with feature extraction of visual images, had the highest density of synapses (mean ± *SD*: 51.74 ± 11.68; Figure 7a). Pons had the lowest density of puncta (mean ± *SD*: 6.26 ± 2.39).

Quantification of PSD95 puncta size across the 20 human brain areas is shown in Figure 7b. PSD95 puncta size reflects PSD size within an individual synapse (Broadhead et al., 2016; Zhu et al., 2018). There was a 1.5-fold difference between the smallest and largest mean PSD95 puncta size. The secondary visual cortex, area BA19, had the largest PSD95 puncta (mean ± *SD*: 0.28 ± 0.03), whereas midbrain had the smallest (mean ± *SD*: 0.188 ± 0.07).

PSD95 puncta intensity across the 20 human brain areas is shown in Figure 7c. Intensity reflects the amount of PSD95 protein in an individual synapse (Broadhead et al., 2016; Zhu et al., 2018). There was a 1.9-fold difference between the highest and the lowest mean intensity in the examined areas. The secondary visual cortex, area BA19, showed the highest intensity of PSD95 puncta (mean ± *SD*: 40.6 ± 9.91), whereas medulla showed the lowest (mean ± *SD*: 21.54 ± 2.65). The deep grey nuclei (thalamus and caudate nucleus) showed greatest variations between individuals. We quantified the synaptic parameters in each of the six layers of 13 neocortical areas and found differences between Brodmann areas and between the layers within these areas (Figure S5). Together, these data show that PSD95 puncta parameters differ between the delineated 20 human brain regions (Figure 7d), with each brain area having a characteristic “signature” of these parameters.

### 3.4 The human PSD95 synaptome architecture is hierarchically organized and similar to that of mouse

**Figure 8:**
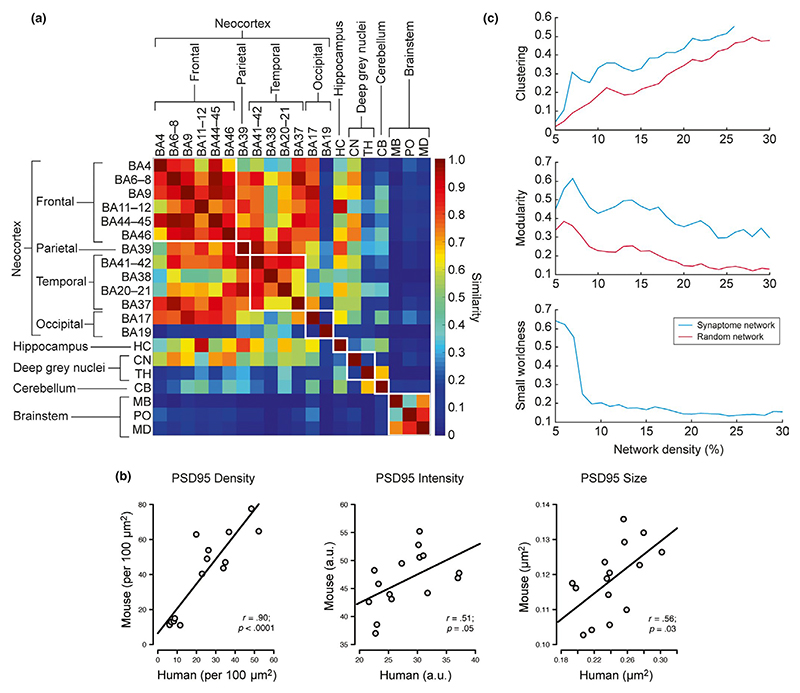
Mapping of PSD95 at whole brain scale. (a) Similarity matrix between pairs of the 20 human brain regions examined (rows and columns). (b) Correlation of PSD95 puncta parameters between human and mouse. Scatterplots show correlations of PSD95 immunofluorescent puncta parameters (density, intensity and size) quantified from 15 brain areas of PSD95-EGFP knock-in mouse and human tissue stained using PSD95 antibody. A positive, linear and significant correlation was found for PSD95 puncta density, intensity and size between the two species. Pearson's product-moment correlation (*r*) and *p* values are provided, based on three male WT mice aged 12 months and three male human controls. (c) PSD95 network topology. Clustering coefficient, modularity, and small-worldness of the PSD95 network and random network

With the quantitative synaptic data in hand, we could now make comparisons between human brain regions. We first examined the similarity of the human brain regions by generating a similarity matrix (Figure 8a), which revealed a number of interesting patterns. All neocortical areas and hippocampus showed high similarity (with the exception of BA19), and these regions were distinct from cerebellum and brainstem structures. BA19 is the visual association area, and it showed the highest PSD95 density, intensity and size. Interestingly, a very similar organization was observed in the mouse brain (Zhu et al., 2018). To test if the PSD95 synaptome architecture of the two species was conserved we correlated the synaptic puncta parameters between the homologous brain regions of the two species and found significant correlations (puncta density, *r* =.90, *p* < .0001; intensity, *r* = .51, *p* = .05; size, *r =* .56, *p* = .03; Figure 8b). We also found a significant correlation coefficient (*r* = .6, *p* = .006) between size and density and a non-significant correlation coefficient (*r* = .4, *p* = .08) between size and intensity.

Synaptome architecture can also be analysed using network methods, and in the mouse, the synaptome shows a small-world network architecture (Zhu et al., 2018). We analysed the synaptome network properties of the 20 human regions: each node in the network represented one region and edges that link nodes were scored by similarity of PSD95 parameters. Three network topological properties, namely clustering coefficient (CC), modularity and small-worldness (Figure 8c), were calculated and showed that the PSD95 network has a higher CC and modularity than a random network with equivalent network complexity. Thus, the human PSD95 synaptome contains a small-world network structure with high small-worldness topology. Together, these findings indicate that the PSD95 synaptome architecture of the human brain shares core features with that of mouse.

## 4 Discussion

Using immunolabelling of PSD95 with high-resolution confocal microscopy and SYNMAP image analysis software, we have examined the distribution of excitatory synapses across 20 regions of the human brain in four individuals. We found that there are populations of PSD95-positive synaptic puncta with different sizes and intensities distributed in each brain region. Comparisons of the mean values of the synaptic parameters of these populations revealed a hierarchical organization of the regions, in which cortical and hippocampal regions shared greatest similarities, whereas cerebellar and brainstem regions had distinct PSD95 synaptome signatures. This is remarkably similar to the PSD95 synaptome organization in the mouse brain (Zhu et al., 2018). Moreover, synaptic puncta density, size and intensity correlated between homologous brain regions in mouse and human, showing evidence of a conserved synaptome architecture. Furthermore, the PSD95 synaptome architecture of both species showed small-world network properties. These data indicate that the synaptome architecture of PSD95-positive excitatory synapses retains invariant features during the 90 million years since humans and mice shared a common ancestor and despite the 1,000-fold difference in brain size. Previous studies have shown that the synapse proteome composition is highly conserved between mouse and human (Bayes et al., 2011, 2012) and that regional diversity in the postsynaptic proteome of the human cortex (Roy, Sorokina, Skene, et al., 2018) and mouse brain shows similar features (Roy, Sorokina, McLean, et al., 2018). Thus, the molecular anatomy of synapse protein composition, synapse diversity and its spatial organization exhibit conserved features between humans and mice.

The present study has a number of limitations. Synapse labelling has been restricted to a single postsynaptic marker of excitatory synapses and as such represents a first step in the task of mapping the human brain synaptome. PSD95 will not, for example, have reported on the diversity of inhibitory synapses. Combining PSD95 labelling with other synaptic markers enables sub-categorization of excitatory synapses (Zhu et al., 2018). Electron microscopy and super-resolution nanoscopy reveal morphological differences in synapses that escape detection with confocal microscopy (Broadhead et al., 2016; Masch et al., 2018), and future studies combining these forms of microscopy will be required for the most comprehensive analyses. A further limitation of this study is that we have not created a fine-scaled PSD95 synaptome map of all the subregions within the 20 regions we have studied. This will require more extensive microscopy, scanning larger areas of brain tissue. We are currently designing experiments and developing technical approaches suitable for mapping all grey matter regions in whole coronal sections of human brain, which is a step towards a brain-wide synaptome atlas of the human.

Post-mortem delay (PMD) could lead to artefacts in the labelling and quantification of synapse proteins using single-synapse resolution methods. Biochemical studies show that human synaptic proteins vary in their rate of degradation with PMD, with PSD95 being relatively stable for at least 24 hr (Bayes et al., 2014; Siew, Love, Dawbarn, Wilcock, & Allen, 2004; Sinclair, Tayler, & Love, 2015). To minimize the effects of PMD, we pre-screened our brain samples using a biochemical assay that measures the integrity of synaptic proteins and selected samples that did not show degradation (Bayes et al., 2014). The image analysis methods we describe in the current study can now be used to examine the impact of PMD on PSD95 puncta parameters.

As high synapse diversity arises from the differential distribution of synaptic proteins and their cognate multiprotein complexes (Grant, 2018, 2019; Zhu et al., 2018), it is likely that a detailed characterization of the synaptome of diverse molecular types of synapses in humans and mice could reveal species-specific differences. Understanding these differences could be extremely important for translational studies from the mouse. Mice carrying mutations in human orthologs that cause schizophrenia and autism have been shown to have altered synapse diversity and synaptome maps (Grant, 2019; Zhu et al., 2018). The availability of synaptomic methods in humans provides an opportunity to identify synaptic pathology in human brain diseases. The availability of a human synaptome atlas for multiple synapse types and comparable atlases in other mammalian species will inform on the function of the human brain and the basis of behaviour in health and disease.

## Supplementary Material

Fig. S1

Fig. S2

Fig. S3

Fig. S4

Fig. S5

Supplementary figure legends

## Figures and Tables

**Table 1:** Details of the human control subjects used in this study

Case ID	SD23/13	SD25/13	SD32/13	SD42/13
Age (years)	53	47	61	46
Gender	Male	Male	Male	Female
Height (m)	1.75	1.83	1.68	1.68
Weight (kg)	112	105	63	102
BMI	36.6	31.4	22.3	36.1
Hx of dementia	No	No	No	No
Smoking	No	Yes	No	Yes
Medication	No	No	Yes	Yes
			Bendrofluazide	Simvastatin
			Amlodipine	Bisoprolol
			Diffundox	Lisinopril
Significant PMH	Depression	Nil	Nil	Hypertension
				Hyperlipidemia
Cause of death	CAT	CAA	HPC	MI

Abbreviations: BMI, Body mass index; CAA, Coronary artery atherosclerosis; CAT, coronary artery thrombosis; HPC, Hemopericardium; Hx of dementia, History of dementia; MI, Myocardial infarction; PMH, Past medical history.

**Table 2:** Neuropathological assessment of human tissue used in this study

Case ID	SD23/13	SD25/13	SD32/13	SD42/13
Brain weight (g)	1,650	1,690	1,270	1,210
PMD (hr)	103	31	99	27
pH	6.1	6.5	6.2	6.5
RIN	5.65	5.30	6.15	4.90
APOE	3/4	3/3	3/4	3/3
HUSPIR	3.75	2.52	3.94	1.83
Thal Aβ	0	0	2	0
Braak NFT	0	0	I	0
CERAD	0	0	I	0
CAA	0	0	0	0

Abbreviations: APOE, Apolipoprotein E; Aβ, Beta-amyloid; CAA, Cerebral amyloid angiopathy; CERAD, Consortium to Establish a Registry for Alzheimer's Disease; HUSPIR, Human synaptic protein integrity ratio; NFT, Neurofibrillary tangles; PMD, Post-mortem delay; RIN, RNA integrity number. The APOE genotypes and HUSPIRs were performed on area BA41/42.

**Table 3:** Human brain areas analysed in this study

Neocortex	1	Motor cortex (BA4)	Layers (I–VI)
	2	Premotor cortex (BA6/8)	Layers (I–VI)
	3	Dorsolateral prefrontal (BA9)	Layers (I–VI)
	4	Orbitofrontal (BA11/12)	Layers (I–VI)
	5	Occipital (BA17)	Layers (I–VI)
	6	Occipital (BA19)	Layers (I–VI)
	7	Inferior temporal (BA20/21)	Layers (I–VI)
	8	Occipitotemporal (BA37)	Layers (I–VI)
	9	Temporal polar (BA38)	Layers (I–VI)
	10	Inferior parietal (BA39)	Layers (I–VI)
	11	Superior temporal (BA41/42)	Layers (I–VI)
	12	Ventrolateral prefrontal (BA44/45)	Layers (I–VI)
	13	Dorsolateral prefrontal (BA46)	Layers (I–VI)
Allocortex	14	Hippocampus (HC)	Cornu ammonis 1 (CA1)
			Cornu ammonis 2 (CA2)
			Cornu ammonis 3 (CA3)
			Cornu ammonis 4 (CA4)
			Dentate gyrus (DG)
Subcortical	15	Thalamus (TH)	
grey nuclei	16	Caudate nucleus (CN)	
Cerebellum	17	Cerebellum (CB)	Granular cell layer (GL)
			Molecular cell layer (ML)
Brainstem	18	Midbrain (MB)	Periaqueductal grey (PAG)
			Substantia nigra (SN)
	19	Pons (PO)	Locus ceruleus (LC)
			Pontine nuclei (PN)
	20	Medulla (MD)	Inferior olivary nucleus (ION)
			Hypoglossal nucleus (CNXII)

Abbreviation: BA, Brodmann area.

## Data Availability

The authors confirm that all data underlying the finding are available and will be shared with the research community upon request.

## Abbreviations

BA: Brodmann area
CA: Cornu Ammonis
CA1-SML: CA1-Stratum Moleculare Lacunosum
CA1-SO: CA1-Stratum Oriens
CA1-SP: CA1-Stratum Pyramidale
CA1-SR: CA1-Stratum Radiatum
CA2-SML: CA2-Stratum Moleculare Lacunosum
CA2-SO: CA2-Stratum Oriens
CA2-SP: CA2-Stratum Pyramidale
CA2-SR: CA2-Stratum Radiatum
CA3-SL: CA3-Stratum Lucidum
CA3-SML: CA3-Stratum Moleculare Lacunosum
CA3-SO: CA3-Stratum Oriens
CA3-SP: CA3-Stratum Pyramidale
CA4: Cornu Ammonis 4
CB: Cerebellum
CN: Caudate Nucleus
CNXII: Hypoglossal Nucleus of Cranial Nerve XII
DG: Dentate Gyrus
DG-PL: DG-Polymorphic Layer
DG-SG: DG-Stratum Granulosum
DG-SM: DG-Stratum Moleculare
EBB: Edinburgh Brain Bank
FFPE: formalin-fixed paraffin-embedded
GCL: Granular cell layer
HC: Hippocampus
IHC: immunohistochemistry
ION: Inferior Olivary Nucleus
LC: Locus Ceruleus
LSCM: Laser Scanning Confocal Microscope
MB: Midbrain
MCL: Molecular cell layer
MD: Medulla
PC: Purkinje cell
PN: Pontine nuclei
PO: Pons
PSD: postsynaptic density
SN: Substantia Nigra
TH: Thalamus
WM: White matter
