# Supplementary figures and images for: A single-synapse resolution survey of PSD95-positive synapses in twenty human brain regions
Source: Eur J Neurosci. Author manuscript; Available in PMC 2024 Feb 28. (PMC7615673; doi:10.1111/ejn.14846)

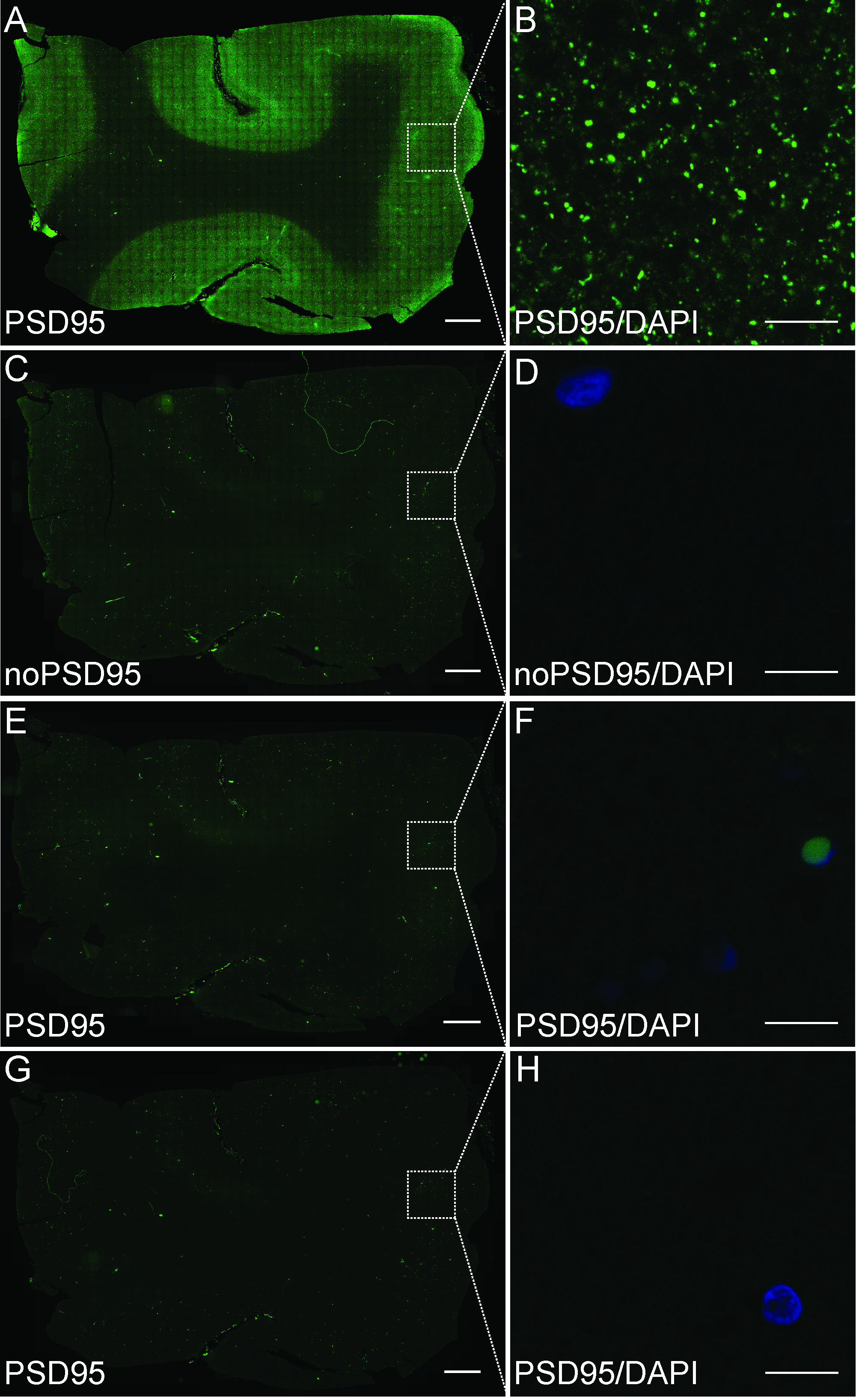

Supplement: Fig. S1 [file EMS194245-supplement-Fig__S1.tif]

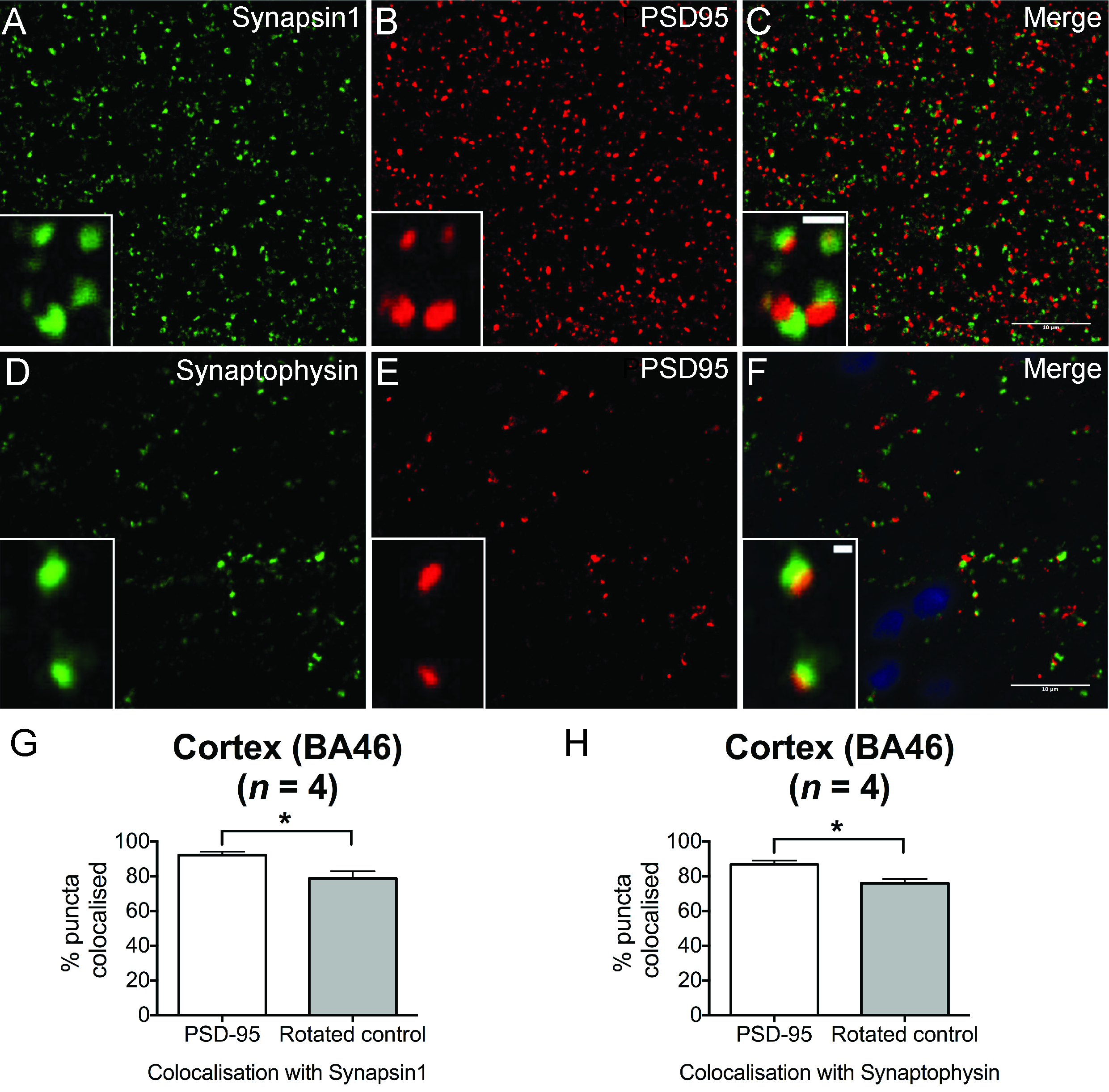

Supplement: Fig. S2 [file EMS194245-supplement-Fig__S2.tif]

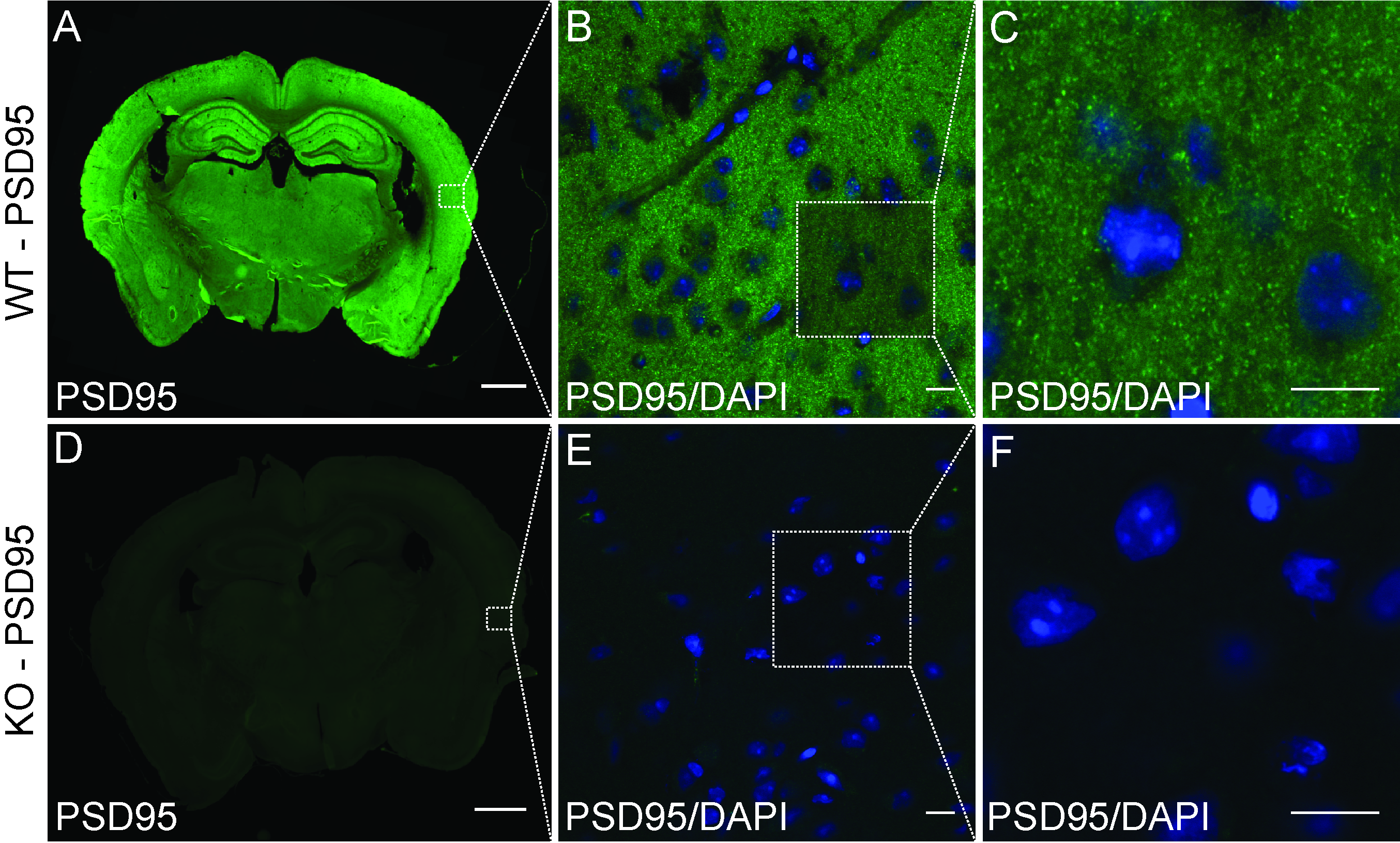

Supplement: Fig. S3 [file EMS194245-supplement-Fig__S3.tif]

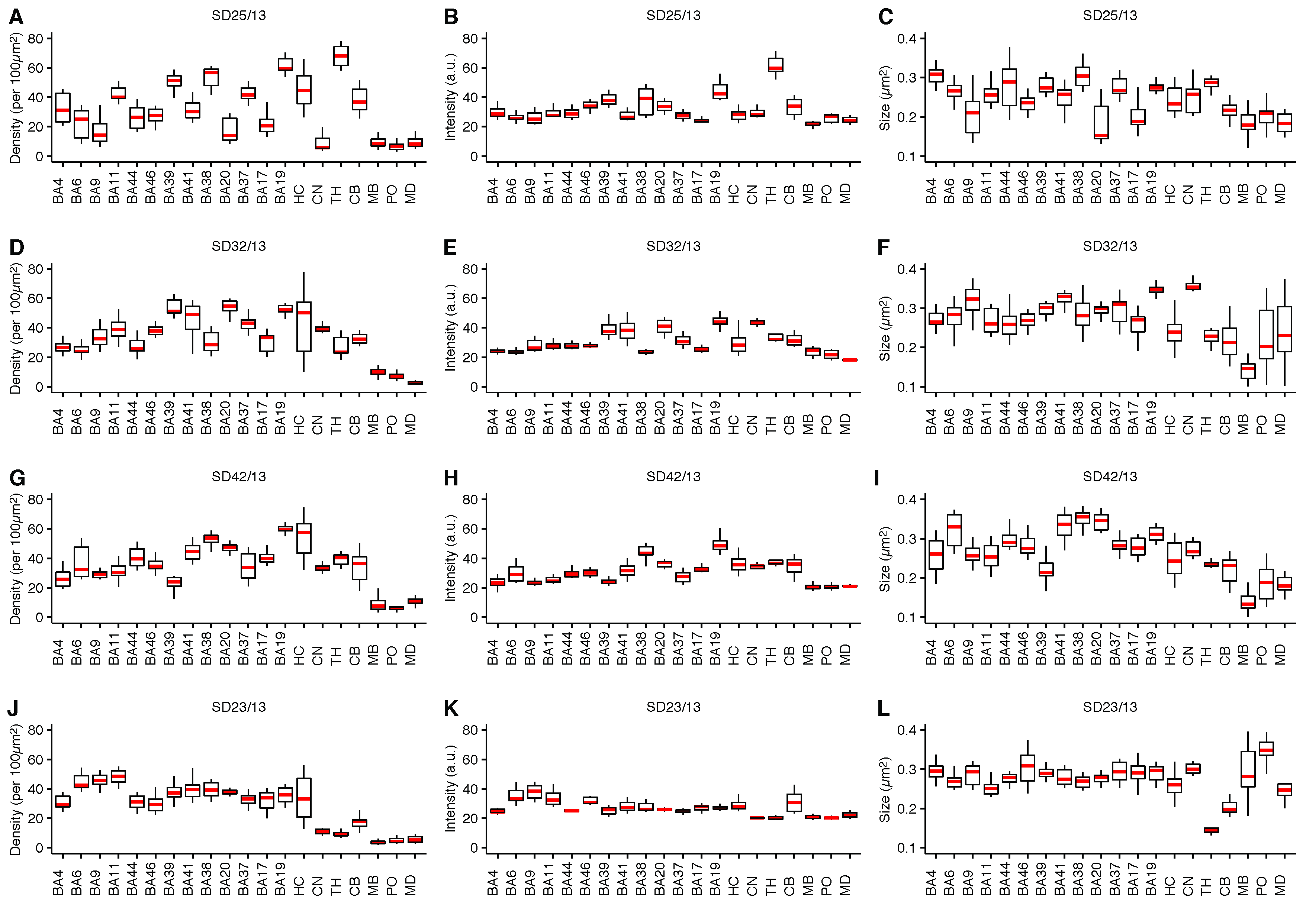

Supplement: Fig. S4 [file EMS194245-supplement-Fig__S4.tif]

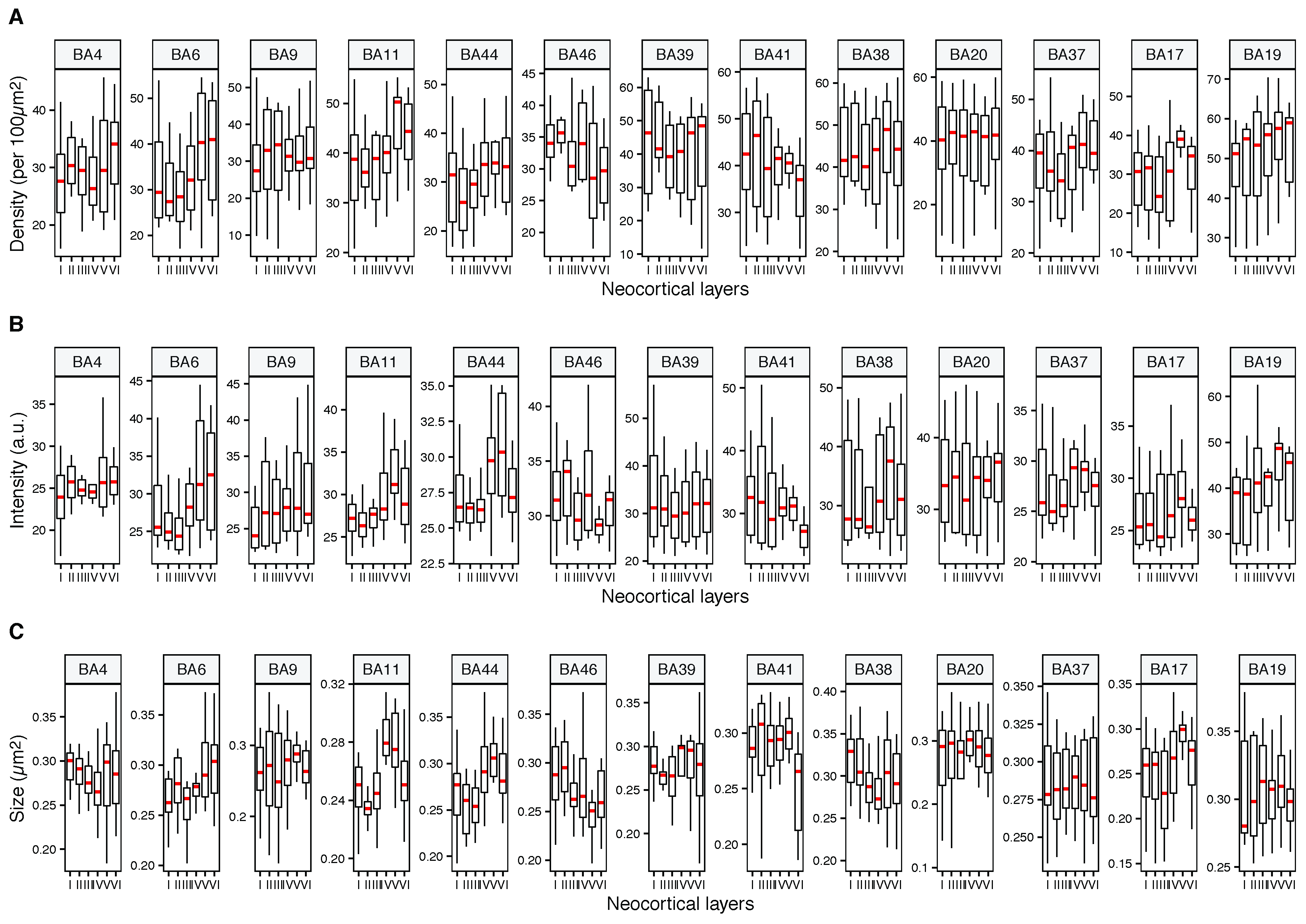

Supplement: Fig. S5 [file EMS194245-supplement-Fig__S5.tif]
