# Supplementary material for: A single-synapse resolution survey of PSD95-positive synapses in twenty human brain regions
Source: Eur J Neurosci. Author manuscript; Available in PMC 2024 Feb 28. (PMC7615673; doi:10.1111/ejn.14846)
Supplement: Supplementary figure legends [file EMS194245-supplement-Supplementary_figure_legends.docx]

# Supplementary Figure Legends

# Figure S1. PSD95 antibody specificity in human post-mortem tissue.

(A, B) PSD95 immunolabelling with primary and secondary antibody in neocortex. Punctate staining shown in (B).

(C, D) PSD95 staining is absent when the primary antibody was omitted.

(E, F) PSD95 staining is absent when the secondary antibody was omitted.

(G, H) PSD95 staining is absent when secondary antibody from another species was used.

PSD95 labelling, green; DAPI labelling, blue.

Scale bars: 1 mm in A, C, E, G; 10 μm in B, D, F, H.

# Figure S2. PSD95 antibody as a reliable excitatory postsynaptic marker.

(A-C) Immunostaining of PSD95 (red) and synapsin 1 (green).

(D-F) Immunostaining of PSD95 (red) and synaptophysin (green).

Synapsin 1 and synaptophysin are markers of presynaptic excitatory synapses and merged images show apposition of pre- and post-synaptic markers.

Scale bars: 10 μm in C, F; 1 μm in insets.

(G, H) Quantification of PSD95-positive puncta colocalisation with synapsin 1 (G) and synaptophysin (H). The rotated control shows the degree of random colocalisation obtained by clockwise rotation of the synapsin 1 (G) or synaptophysin (H) images. The colocalisation with either presynaptic marker is significantly higher than expected by chance. All images obtained from superficial cortical layers. Data are mean ± SD; *p ≤ 0.05, Mann-Whitney U-test.

# Figure S3. PSD95 antibody staining in wild-type and *Psd95* knockout mice.

(A-C) Immunostaining of PSD95 in a wild-type (WT) mouse.

(D-F) Immunostaining of PSD95 is abolished in *Psd95* knockout (KO) mouse.

(A, D) low magnification (20x).

(B, C, E, F) high magnification (63x).

PSD95 labelling, green; DAPI labelling, blue.

Scale bars: 1 mm in A, D; 10 μm in B, C, E, F.

# Figure S4. PSD95 puncta parameter distribution in brain areas of four subjects.

Cases:

(A-C) SD25/13

(D-F) SD32/13

(G-I) SD42/13

(J-L) SD23/13.

PSD95 puncta parameters:

(A, D, G, J) PSD95 density (per 100 μm^2^)

(B, E, H, K) PSD95 intensity (a.u.)

(C, F, I, L) PSD95 size (μm^2^).

Within each boxplot, horizontal lines show the median (in red); box limits indicate the interquartile range (IQR) between 1^st^ quartile range = 25^th^ percentile and 3^rd^ quartile range = 75^th^ percentile; and whiskers are defined by Tukey and extend to data points that are less/more than 1.5x IQR away from the 1^st^/3^rd^ quartile.

BA, Brodmann area; HC, hippocampus; TH, thalamus; CN, caudate nucleus; CB, cerebellum; MB, midbrain; PO, pons; MD, medulla.

**Figure S5. Comparison of PSD95 synaptic puncta parameter distribution for neocortical layers I to VI of 13 Brodmann areas for four human subjects.**

PSD95 puncta density (per 100 μm^2^) (A), intensity (a.u.) (B) and size (μm^2^) (C) for subjects SD25/13, SD32/13, SD42/13 and SD23/13. Horizontal red lines indicate the median; box limits indicate the interquartile range (IQR) between 1^st^ quartile range = 25^th^ percentile and 3^rd^ quartile range = 75^th^ percentile; and whiskers are defined by Tukey and extend to data points that are less/more than 1.5x IQR from the 1^st^/3^rd^ quartile. BA, Brodmann area.
